# The effect of vitamin D on the severity of dysmenorrhea and menstrual blood loss: a randomized clinical trial

**DOI:** 10.1186/s12905-023-02284-5

**Published:** 2023-03-27

**Authors:** Azam Amzajerdi, Maryam Keshavarz, Elham Ghorbali, Sally Pezaro, Fatemeh Sarvi

**Affiliations:** 1 School of Nursing and Midwifery, Tehran University of Medical Sciences, Tehran, Iran; 2 Department of Midwifery and Reproductive Health, School of Nursing and Midwifery, Reproductive Sciences and Technology Research Center, Department of Midwifery and Reproductive Health, Iran University of Medical Sciences, Rashid Yasemi St., Valiasr St, Tehran, 1996713883 Iran; 3 Centre for Healthcare Research, Coventry University, Coventry, UK; 4 The University of Notre Dame, Fremantle, Australia; 5 Department of Public Health, School of Health, Larestan University of Medical Sciences, Shiraz, Fars, Iran

**Keywords:** Menstrual blood loss, Primary dysmenorrhea, University students, Vitamin D

## Abstract

**Background:**

Primary dysmenorrhea is considered as one of the women’s main problems during reproductive age. The present study aimed to investigate the effect of vitamin D on the severity of dysmenorrhea and menstrual blood loss.

**Methods:**

This double-blind, randomized, placebo-controlled trial, was performed on 84 single female college students between 18 and 25 years old who living in dormitories. Students with primary dysmenorrhea and vitamin D deficiency were divided into experimental (n = 42) and control (n = 42) groups. Five days before the putative beginning of their next menstrual cycle, the experimental group received 300,000 IU vitamin D (50,000 IU, two tablets every 8 h), and the control group received a placebo (oral paraffin). The effects of the supplement on the severity of dysmenorrhea and menstrual blood loss were evaluated one cycle before and during two successive cycles. Using the visual analog scale (VAS), verbal multidimensional scoring system (VMS), and pictorial blood assessment chart (PBLAC) questionnaires. Fisher’s exact, Chi-square, independent sample t-test and repeated measurements were used.

**Results:**

In total, 78 of the 84 students completed the study (39 students per group). The intervention resulted in a significant reduction in the mean scores of both the VAS and VMS in the experimental group, in the first and second menstrual cycles (p < 0.001, p < 0.001, respectively), but not in the means score of PBLAC. Mefenamic acid consumption at the first and second menstruation period, in the experimental group was lower than the control group (p = 0.009, p < 0.001, respectively).

**Conclusions:**

The results indicate that vitamin D supplementation could decrease the severity of primary dysmenorrhea and the need to consume pain-relief medications. Contrariwise vitamin D supplementation had no significant effect on menstrual blood loss.

**Trial registration:**

This trial was registered in the Iranian Registry of Clinical Trials with code IRCT201305212324N on 18/1/2014. URL of registry: https://en.irct.ir/trial/1964.

**Supplementary Information:**

The online version contains supplementary material available at 10.1186/s12905-023-02284-5.

## Background

Primary dysmenorrhea is defined as pain occurring with menses in the absence of pelvic pathology [1]. It leads to workplace and school absences, reduces ones quality of life and general wellbeing, and is associated with high health and social-economic costs [2], affects up to 25% of all menstruating women, with prevalence ranges from 20 to 90% [1]. The prevalence of primary dysmenorrhea was 73.27% in Iran [3].

Non-steroidal anti-inflammatory drugs (NSAIDs) and oral contraceptive pills (OCPs) are recommended as first-line medication for pain management. However, complications and dissatisfaction with them are prominent [4]. As such, alternative solutions may be useful in pursuit of improved outcomes for women and girls. Due to the diverse functions of vitamin D in the body, the attention to its use in medicine has increased [5]. A meta-analysis of seven observational studies involving 2420 patients also concluded that low levels of vitamin D concentrations may be associated with other pain conditions [6]. In some studies the effect of weekly administration of high dose of vitamin D in reducing menstrual pain has been reported [7–9]. In one study, a single dose of 300,000 IU vitamin D reduced the severity of dysmenorrhea in the first month [10], but in another study prescription of 300,000 IU vitamin D five days before the beginning of the menstrual cycle, for three consecutive cycles, showed a positive effect only in the second and third months after the intervention [11]. Further research is required in this area, particularly where rates of primary dysmenorrhea is high.

Vitamin D deficiency is prevalent in most countries [12], has become one of the most important health issues in the world [13]. A systematic review and meta-analysis of 48 studies in Iran identified 18,531 individuals with vitamin D deficiency. The prevalence of vitamin D deficiency among male, female, and pregnant women was estimated to be 45.64%, 61.90%, and 60.45%, respectively [14]. Thus, further research in this area may usefully be conducted in Iran specifically.

The active form of vitamin D can reduce prostaglandin production in the endometrium and limit its biological activity by affecting prostaglandin receptors [15]. It may also exert anti-inflammatory effects through various pathways [16]. It remains unclear whether vitamin D is therapeutic for reducing menstrual flow. In one study, vitamin D supplementation reduced the number of subjects with heavy menstrual flow, yet this was not statistically significant [7]. Considering the effect of vitamin D in reducing prostaglandins, it is expected that vitamin D would also be effective in reducing menstrual bleeding. In light of the above, the present study was designed based on a hypothesis that prescription high-dose vitamin D would significantly decrease dysmenorrhea and blood loss in two consecutive cycles.

## Methods

### Study design

This is a double-blind, randomized, placebo-controlled trial with two parallel groups. The study samples were female college students with primary dysmenorrhea, who living in Tehran University of Medical Sciences (TUMS) dormitories in Tehran, Iran.

### Participants

Participants were healthy, Iranian, and single female college students. The inclusion criteria included: age 18 to 25 years old, BMI ≤ 30 (kg/m2), regular menstrual cycles of 21–35 days with menstrual period of 3–7 days, experience at least four consecutive painful periods in the past six months with the pain starting a few hours before or just after the onset of bleeding, vitamin D deficiency (25[OH]D serum level ≤ 30 ng/mL), normal serum calcium (8.6–10.3 mg/dL), no one of the following conditions: a history of underlying disease causing secondary dysmenorrhea (e.g. endometriosis or and adenomyosis), undertaking regular exercise, exposure to recent stressful events (past 3 months), using oral contraceptives and/or hormonal drugs (past 3 months), using any medications containing or interacting with calcium and/or vitamin D (past 6 months), special dietary (e.g. vegetarian), smoking or engaging in alcohol consumption. Participants were excluded if they had no menstruation during the intervention period, used oral contraceptives and/or other hormonal drugs along with non-pharmacological methods or pain relief supplements, smoking or engaging in alcohol consumption through the duration of the intervention period, vomiting 2 h after supplement consumption, or unwillingness to continue the study.

### Sample size

Sample size was obtained as 66 students based on previous studies [10], with considering the probability of confidence coefficient as 95% and the mean (SD) pain score as 5.45 (1.79) for control group and the mean (SD) pain score as 3.7 (1.34) for experimental group. Taking into account an estimation of 10% drop out, finally 84 (42 in each group) students included in the study. A study with such a sample size would have a power of 90% at 5% significant level and an effect size of 1.5.

### Randomization

Among the TUMS dormitories, two dormitories were randomly selected. First, a list of students who met the inclusion criteria was prepared, then numbers were assigned to participants. Using a random numbers table, eighty-four single female college students were enrolled in the study. Numbers 1 to 84 were divided into two groups by Randomizer statistical program. By the pharmaceutical company, one group of numbers was recorded on vitamin D packages, and the next group of numbers was recorded on placebo packages.

### Study instruments

The VAS was used to measure menstruation pain. It is a horizontal scale with the descriptors 0: no pain and 10: worst possible pain [17]. In the same way, scores from the VAS are categorized as mild: 1–3; moderate: 4–7; severe: 8–10. Higher scores indicate increased levels of pain [18]. The test-retest reliability of VAS was 0.89 [19].

The VMS was also used to assess students’ perception of menstrual pain severity and its effect on their daily activities. Items were scored, using a four-point Likert scale ranging from no symptoms to severe symptoms (i.e., none, mild, moderate, and severe) [18]. The reliability of VMS was examined using the test-retest method, and the correlation coefficient was 0.80 [19]. The PBLAC was used to measure the amount of menstrual bleeding [20]. In a systematic review of methods to measure menstrual blood loss, the sensitivity and specificity of PBAC were reported at 58–99%, and 75–89%, respectively [21]. In the current study, Cronbach’s alpha coefficient was found for VAS, VMS, and PBLAC to be 0.89, 0.90, and 0.88 respectively.

### Intervention and outcomes

The procedure was explained to the participants who met the inclusion criteria. All participants signed informed written consent. Subsequently, 5 ml of venous blood was taken to determine serum levels of 25(OH)D and calcium. These blood samples were then transferred to a laboratory affiliated with TUMS, centrifuged (2,500 rpm for 5 min), serum separation was performed and the samples were immediately frozen at -70 °C. ELISA and a kit from EUROIMMUN, a UK manufacturing company measured 25(OH)D. This kit’s mean internal and external variation coefficient was 4.9 and 7.8%, respectively, and the optimum level of 25(OH)D, 30–50 ng/ml was determined based on the information of this kit. Cresolphthalein complexone (CPC) and the Kate method were used to measure serum calcium levels by a Pars Company in Iran. This kit’s mean internal and external variation coefficient was 1.45% and 1.66%, respectively, and the desired calcium level was 8.6–10.3 mg/dl, based on the data of this kit.

As guided by the Lasco study (2012) [9], five days prior to the putative beginning of their next menstrual cycle, the experimental group received 300,000 IU of vitamin D (six tablets of 50,000 IU). The control group received six oral placebo doses, containing oral paraffin. Zahravi Iran Pharmaceutical Company made the vitamin D and placebo the same shape and size. These were pre-coded by a consultant pharmacist and remained unknown to the researcher and samples. Participants were permitted to use mefenamic acid (250 mg) and recorded the number of it taken per day prior to the intervention and during the first and second menstrual cycles following the intervention. A checklist of symptoms associated with vitamin D including anorexia, lethargy, nausea, and vomiting was delivered to participants to complete after the intervention period. One month after receiving the medication and placebo, the subjects were re-evaluated for serum 25(OH)D and calcium levels. Participants completed the visual analog scale (VAS), verbal multidimensional scoring system (VMS), and pictorial blood assessment chart (PBLAC) questionnaires during the menstruation period before the intervention, and also in the first and second cycles after the intervention.

### Statistics analysis

The Skewness test was used to assess the normality of quantitative variables. Descriptive statistics, independent sample t-test, and repeated measurements were utilized for to examine continuous quantitative variables. Meanwhile, Fisher’s exact and Chi-square tests were used to examine associations between variables. Repeated measurements were used to compare the mean score of VAS, VMS, PBLAC, and mefenamic acid consumption between the two groups over time. In addition, the Greenhouse-Geisser test was used in cases where the sphericity of the test was not assumed. SPSS version 16 was used for the data analysis, and the p < 0.05 was considered statistically significant.

## Results

**Fig. 1:**
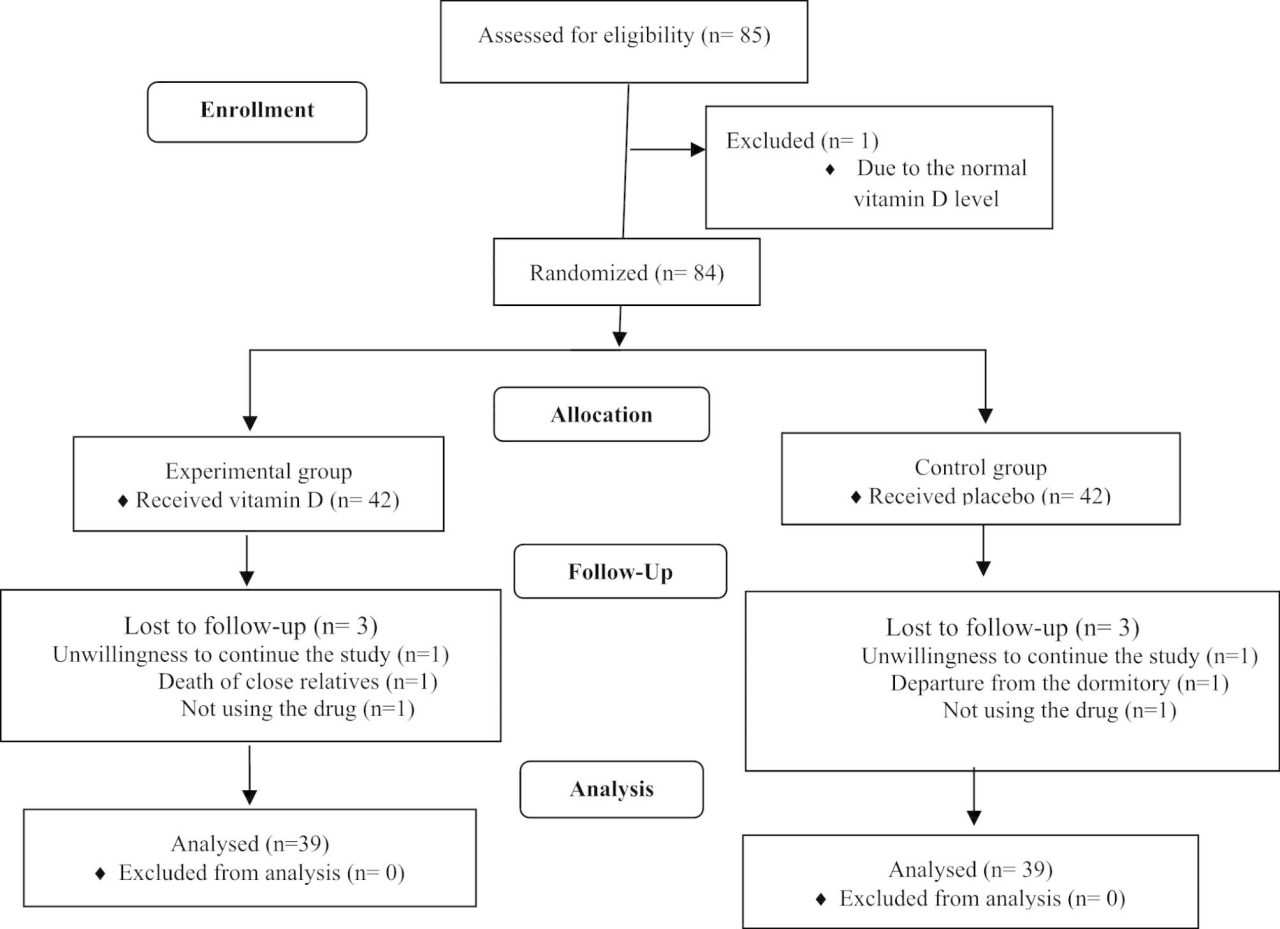
CONSORT 2010 Flow Diagram

Of the 84 participants recruited for this study, 39 students in each group, completed the three-month study. Excluded cases represent in Fig. 1.

Findings showed no significant difference between the two groups concerning their socio-demographic and menstruation characteristics (Table 1). A significant reduction in the mean score of VAS and VMS was observed in the experimental group compared to the control group during the first and second months of the intervention period, but not in the mean score of the PBLAC (Table 2). One month after the intervention period, serum 25(OH)D level significantly improved in the experimental group compared to the control group. No significant difference was observed in serum calcium levels (Table 3). Mefenamic acid consumption during the first and second menstruation periods, in the experimental group, was lower than in the control group, and significantly decreased in the experimental group over time (Table 4).

**Table 1:** Socio-demographic data and menstruation profile in the two groups.

Variables	Experimental group (n = 39)	Control group (n = 39)	P-value
**Age** (year)	21.80 ± 2.02	21.40 ± 1.69	0.367^a^
**BMI** (Kg/m2)	21.20 ± 2.68	21 ± 2.20	0.157^a^
**Economic Status** (Good)	17 (43.6%)	12 (30.8%)	0.169^b^
**Skin color** (Light)	27 (69.2%)	26 (66.7%)	0.808^b^
**Duration of exposure to direct sunlight**			
< 50 min	34 (87.2%)	29 (74.4%)	0.342^c^
50–100 min	4 (10.3%)	5 (12.8%)	
100–150 min	1 (2.5%)	4 (10.3%)	
> 150 min	0	1 (2.5%)	
**Age at menarche** (year)	12.80 ± 1.21	13.20 ± 1.19	0.094^a^
**Bleeding duration** (day)	6.10 ± 1.11	6.00 ± 0.98	0.748^a^
**Menstrual cycle duration**(day)	28.05 ± 2.77	28.51 ± 3.24	0.501^a^
**Dysmenorrhea duration** (day)	2.30 ± 0.70	2.60 ± 0.98	0.192^a^

Data presented as mean ± Standard Deviation or number (percentage)

BMI: Body mass index

^a^ Independent sample t-test

^b^ Chi square

^c^ Fisher exact test

**Table 2:** The mean score of VAS, VMS, and PBLAC scores in the two groups.

	Experimental group (n = 39)	Control group (n = 39)	P-value
**VAS**			
Pre-intervention	6.71 ± 2.25	6.64 ± 2.46	0.862^a^
First month post-intervention	5.33 ± 2.39	6.53 ± 2.30	< 0.010^a^
Second month post-intervention	3.92 ± 2.36	6.79 ± 2.17	< 0.001^a^
P-value^b^			0.001^b^
**VMS**			
Pre-intervention	2.00 ± 0.45	2.00 ± 0.64	1^a^
First month post-intervention	1.40 ± 0.67	2.00 ± 0.64	< 0.001^a^
Second month post-intervention	0.94 ± 0.55	2.05 ± 0.60	< 0.001^a^
P-value^b^			< 0.001^b^
**PBLAC**			
Pre-intervention (cc)	74.60 ± 55.20	89.10 ± 71.10	0.391^a^
First month post-intervention (cc)	74.40 ± 74.10	89.80 ± 77.60	0.439^a^
Second month post-intervention (cc)	79.80 ± 79.80	97.10 ± 82.10	0.350^a^
P-value^b^			0.329^b^

Data presented as mean ± Standard Deviation

^a^ Independent sample t-test

^b^ Greenhouse-Geisser

**Table 3:** Serum vitamin D and calcium levels before and one month after the intervention in the two groups.

	Experimental group (n = 39)	Control group (n = 39)	P-value^a^
**25(OH)D** (ng/ml)			
Pre- intervention	5.10 ± 3.310	6.60 ± 5.63	0.110
First month post-intervention	30.63 ± 5.43	9.73 ± 4.72	< 0.001
**Calcium** (mg/dl)			
Pre-intervention	9.20 ± 0.41	9.08 ± 0.34	0.187
First month post-intervention	9.02 ± 0.36	9.02 ± 0.28	0.945

Data presented as mean ± Standard Deviation

^a^ Independent sample t-test

**Table 4:** Using of mefenamic acid in the two groups.

	Experimental group (n = 39)	Control group (n = 39)	P-value^a^
Pre-intervention	2.41 ± 2.18	1.89 ± 1.33	0.516
First month post-intervention	1.35 ± 1.51	1.94 ± 1.27	< 0.010
Second month post-intervention	0.74 ± 1.44	1.89 ± 1.46	< 0.001
P-value^b^			< 0.001

Data presented as mean ± Standard Deviation

^a^ Independent sample t-test

^b^ Greenhouse-Geisser

## Discussion

The present study aimed to investigate the effect of 300,000 IU single-dose vitamin D supplement (50,000 IU, two tablets every 8 h) on the severity of primary dysmenorrhea and menstrual blood loss in Iranian university students. The results revealed that one and two months after the prescription of vitamin D, the mean score and severity of primary dysmenorrhea, also mefenamic acid consumption in the experimental group were statistically less than those in the control group. Still, the intervention had no significant effect on menstrual blood loss. Moreover, one month after the intervention, serum 25(OH)D level significantly increased in the experimental group.

Some studies have also demonstrated the influential role of vitamin D in primary dysmenorrhea. One study investigated the effect of a single oral dose of vitamin D (300,000 IU), five days before the putative beginning of their next menstrual cycle on primary dysmenorrhea. The results indicated that intervention significantly reduced primary dysmenorrhea and NSAID consumption compared with the placebo group over the two months. [9]. The method of vitamin D administration in our study was similar to this study, but in our study for better tolerance of the vitamin D, two tablets (50,000 IU) every 8 h were used separately. In another study a single oral dose of 300,000 IU of vitamin D, five days before the menstruation for three consecutive cycles decreased pain severity significantly in the intervention group in the second and third months after the intervention, but not in the first months [10].

The prescription of 50,000 IU of vitamin D, weekly for eight consecutive weeks reduced pain intensity, the number of days with pain, and the need for consuming pain-relief medications in 116 female students aged 18 to 32 years with primary dysmenorrhea and vitamin D deficiency [9]. A weekly dose of 50,000 IU of vitamin D for 8 weeks in sixty women aged 18–30 years with primary dysmenorrhea [8], also weekly intake of 50,000 IU oral vitamin D for nine weeks in adolescent girls range 12–18 years reduced the severity of dysmenorrhea [7]. In reviewing the above studies, it appears that high doses of vitamin D have a positive effect on reducing menstrual pain, without any observed side effects. According to the available studies, high dose vitamin D supplementation, in single or multiple doses, can reduce the severity of primary dysmenorrhea.

One study showed that the daily prescription of 5,000 IU vitamin D with 1,000 IU calcium from the 15th day of cycle until menstrual pain disappeared in the following cycle had no significant effect on reducing menstrual pain [20]. In a systematic review of 17 studies and 2,828 women, ages 12 to 30 years old, vitamin D supplementation in any form or dose could effectively reduce the severity of primary dysmenorrhea [21]. These conflicting results can be the basis for the design of the next research.

Vitamin D receptors (VDRs) are distributed in the ovary and uterus [22], so vitamin D has an active role in female reproductive system [23]. Its receptor also plays a significant role in regulating steroid hormones in the female reproductive system [15]. Studies suggest a sufficient serum level of the vitamin D has positive effects on ovary function and regulation of menstrual cycles [24]. Prostaglandin metabolites increase vasoconstriction and myometrial contractions causing uterine ischemia in the uterus and pain [25]. Vitamin D may have a positive effect on dysmenorrhea with a variety of mechanisms. In the endometrium, 1,25-dihydroxyvitamin D (1,25[OH]_2_D), the bioactive form of vitamin D, decreases prostaglandin synthesis by suppressing expression of cyclooxygenase-2, and increases prostaglandin inactivation by up-regulating 15-hydroxyprostaglandin dehydrogenase. In addition, 1,25(OH)_2_D down-regulates prostaglandin receptor expression. 1,25(OH)_2_D may also exert anti-inflammatory effects through other pathways [16]. The liver converts all available cholecalciferol into 25(OH)D. The major source of vitamin D in the body is this metabolite, which has a half-life of at least three months [26].

Studies on the impact of vitamin D on menstrual bleeding are limited. In one study, low-dose vitamin D (5,000 IU vitamin D) had no significant effect on menstrual blood loss [20]. Furthermore, high dose vitamin D (50,000 IU) weekly for nine-week had no effect on the heavy menstrual flow [7]. Similarly, in our study, high dose of vitamin D did not reduce menstrual bleeding in the experimental group. Future randomized controlled clinical trials with a greater sample size, longer duration of treatment, different doses of vitamin D and more extended follow-up periods are therefore required to confirm the efficacy of vitamin D in treating menstrual blood loss.

The point to be considered in the current study is that the high doses of vitamin D reduced menstrual pain but did not affect menstrual bleeding. Yet vitamin D may be more effective in people who have severe bleeding. Also, the effect of vitamin D on menstrual pain with other mechanisms should be investigated. Considering the effect of vitamin D supplement in women with and without vitamin D deficiency may answer this ambiguity to some extent. Therefore, future studies in this area may usefully be designed differently to explore this possibility. Strong points of the present study include the measurement of vitamin D levels before and after vitamin D administration and investigation of the effect of high dose vitamin D on dysmenorrhea and the blood loss simultaneously.

## Conclusions

Based on the study findings, over two follow-up periods, a high dose of vitamin D could improve pain intensity and decrease the need for using NSAIDs in women with primary dysmenorrhea and vitamin D deficiency. Vitamin D deficiency is common among those living in Iran. As such, by using supplementation, the amount of vitamin D deficiency and painkillers’ side effects, may be reduced. Due to the acceptability, availability, and cost-effectiveness of this treatment, it could also be used as effective management for dysmenorrhea. More clinical trials with a greater sample size, longer treatment duration, and follow-up period are required to further confirm the efficacy of vitamin D in reducing menstrual blood loss.

## Electronic supplementary material

Below is the link to the electronic supplementary material.

Additional File: Highlights

## Data Availability

The datasets generated in the current study are available from the corresponding author.

## Abbreviations

BMI: Body mass index
CPC: Cresolphthalein complexone
ELISA: Enzyme-linked immunosorbent assay.
IU: International units
NSAIDs: Non-steroidal anti-inflammatory drugs
OCPs: Oral contraceptive pills
PBLAC: Pictorial blood assessment chart
VAS: Visual analog scale
VDRs: Vitamin D receptors
VMS: Verbal multidimensional scoring system
