# Supplementary material for: The effect of vitamin D on the severity of dysmenorrhea and menstrual blood loss: a randomized clinical trial
Source: BMC Womens Health. 2023 Mar 27;23:138. doi: 10.1186/s12905-023-02284-5 (PMC10045437; doi:10.1186/s12905-023-02284-5)
Supplement: Supplementary file 1 — Additional File: Highlights [file 12905_2023_2284_MOESM1_ESM.docx]

Highlights

- Vitamin D is an effective method to relieve pain in primary dysmenorrhea.
- Vitamin D could decrease the need to consume pain-relief medications.
- Vitamin D had no significant effect on menstrual blood loss.
